# Evaluation of the relationship between body composition measurements and metabolic syndrome severity score in obese individuals

**DOI:** 10.1097/MD.0000000000041943

**Published:** 2025-03-21

**Authors:** Yilmaz Sezgin, Serkan Çoşkun, Yunus Aslan, Seda Şimşek, Sümeyye Kaya

**Affiliations:** a Family Medicine Clinic, Trabzon Medicine Faculty, Turkish Health Science University, Trabzon, Turkey; b Lord North Ward, Maidstone Hospital, Kent, England.

**Keywords:** body mass index, fat mass, metabolic syndrome severity score, muscle mass

## Abstract

Metabolic syndrome, characterized by a combination of obesity, hypertension, and metabolic abnormalities (lipid and glucose dysregulation), significantly increases the risk of cardiovascular diseases. This study aimed to explore the association between body composition and severity of metabolic syndrome in obese individuals. This cross-sectional study analyzed data from 180 individuals who sought treatment at an obesity center. Key variables included body mass index (BMI), fat mass, muscle mass, and the metabolic syndrome severity score. Logistic regression was used to evaluate the relationship between body composition parameters and metabolic syndrome severity score. Of the 180 participants, 92.8% were female and 7.2% were male. Individuals with higher metabolic syndrome severity scores had significantly higher age, BMI, fat mass, muscle mass, and fat and muscle ratios. Logistic regression analysis revealed that each 1-unit increase in BMI was associated with a 1.288-fold increase in the risk of a higher metabolic syndrome severity score. However, fat and muscle mass as well as their percentages were not significantly associated with the score. BMI emerged as a key factor influencing the severity of metabolic syndrome in obese individuals, whereas other body composition parameters did not show a significant relationship. These findings highlight the importance of BMI in the management of obesity and metabolic syndrome, and underscore the need for further research with larger sample sizes.

## 1. Introduction

Obesity is a global health problem that has a profound negative impact on overall health. One of the major pathologies associated with obesity is metabolic syndrome, a rapidly growing health concern in both developed and developing countries.^[1]^ The rise in metabolic syndrome, driven by lifestyle changes in these countries, has led to an increased incidence of cardiovascular diseases (CVD).^[2]^

The prevalence of metabolic syndrome (MetS) is reported to be approximately 22% among adults.^[3]^ Metabolic syndrome is characterized by a combination of obesity, hypertension, and abnormalities in lipid and glucose metabolism, all of which predispose individuals to CVD development.^[4]^ Each component of MetS represents an independent risk factor for CVD, and its combined presence significantly increases the likelihood of cardiovascular complications, including microvascular dysfunction, coronary atherosclerosis, cardiac dysfunction, myocardial infarction, and heart failure.^[5]^ A recent cohort study further demonstrated that MetS independently increases the risk of CVD irrespective of all-cause mortality.^[6]^

Understanding the relationship between body composition and metabolic syndrome, particularly using the metabolic syndrome severity score, can offer valuable insights into combating this syndrome, which is recognized as a significant independent risk factor for cardiovascular events. This study aimed to assess the role of body mass index (BMI), muscle mass, and fat mass in obese individuals and examine their association with the metabolic syndrome severity score, while accounting for additional factors such as age, sex, socioeconomic status, chronic diseases, and physical activity levels.

## 2. Materials and methods

### 2.1. Study design

This cross-sectional study was approved by the Turkish Health Sciences University Trabzon Faculty of Medicine scientific research ethics committee (approval dates and numbers: January 16, 2024, and 2024/01). Informed consent regarding medical interventions is obtained from patients when they first apply to the obesity center. In addition, since the data were collected from their medical records, informed consent was not required.

### 2.2. Population selection

The sample size was calculated using the formula: n = N (*t*² × *p* × *q*)/[*d*² ×  (N − 1) + *t*² × *p* × *q*], yielding a minimum required sample size of 160 participants (N = 400, *p* = 22, *q* = 1 − *p*, *t* = 1.96; *d* = 0.05). The study included patients diagnosed with obesity according to the World Health Organization criteria, who applied to Trabzon Kanuni Training and Education Hospital’s obesity center between January 2021 and January 2024, with complete and accessible data in their medical records. Patients whose records were unavailable as well as those under 18 or over 65 years of age were excluded. Finally, data from 180 individuals were included in the study. The body composition of the participants, including the BMI, muscle mass, and fat mass, was recorded. Additionally, data necessary for calculating the metabolic syndrome severity score (MetS-S), such as age, race, sex, BMI, triglycerides, HDL cholesterol, systolic blood pressure, and glucose levels, were entered into the SPSS software.

Body composition was measured by the bioelectrical impedance method. Bioelectrical impedance analysis is a tissue measurement method based on generating information about tissues by examining the resistance encountered by weak electrical currents as they pass through the body tissues. This method is used to determine the water, fat, muscle, and bone ratios of a person’s body structure in a more meaningful manner than BMI.

The BMI-based MetS-S was utilized to assess the risk of metabolic syndrome. The MetS-S was calculated using the online “MetS Severity Score Calculator” (https://metscalc.org/) developed by Gurka and DeBoer. The equations used to calculate MetS-S were derived from the US NHANES study and included variables such as age, race, sex, BMI, blood glucose, triglycerides, high-density lipoprotein cholesterol, and systolic blood pressure.^[7]^ In this study, MetS-S zero (MetS-Sz), which ranges from negative to positive infinity and is one of the 2 parameters calculated for MetS-S, was used.^[8]^ Participants were classified into two groups based on MetS-Sz variables: low-risk and high-risk. Additionally, the percentage of fat and muscle mass was derived from the measured body fat and muscle mass values to provide a more comprehensive analysis of the body composition.

### 2.3. Statistical analysis

All statistical analyses were performed using the IBM SPSS Statistics (V25). Statistical significance was set at *P* < .05. Categorical data, numbers and percentages, and numerical data are expressed as averages and standard deviations. The distribution of demographic data was analyzed by frequency tests, comparison of categorical data by chi-square test and Fisher’s exact test, and comparison of numerical data by independent sample *t* test. The enter model was used in the binary logistic regression test to evaluate the effect of body composition on metabolic syndrome severity score. Skewness and kurtosis analyses were performed to confirm that the data had a normal distribution.

## 3. Results

A total of 180 individual, 167 (92.8) female and 13 (7.2) male, were included in the study. Demographic data and body composition values of the 180 participants included in the study are presented in Table 1.

**Table 1:** Distribution of demographic and body composition data.

Characteristics of participants		
Age (mean ± SD)		45.58 ± 11.73
BMI (mean ± SD)		40.24 ± 6.86
Fat mass (mean ± SD)		43.45 ± 11.21
Muscle mass (mean ± SD)		58.02 ± 8.68
Fat percentage (mean ± SD)		41.13 ± 4.65
Muscle percentage (mean ± SD)		43.46 ± 11.23
MetS-Sz (mean ± SD)		0.73 ± 0.71
MetS-S% (mean ± SD)		72.24 ± 19.76
Gender n (%)	Female	167 (92.8)
	Male	13 (7.2)
Smoking n (%)	Yes	18 (10.0)
	No	162 (90.0)
Chronic disease n (%)	Yes	123 (68.3)
	No	57 (31.7)
Physical activity n (%)	Use	37 (20.6)
	Not use	143 (79.4)
Marital status n (%)	Marriage	156 (86,7)
	Single	24 (13.3)

SD = standard deviation.

Of the 180 individuals, 26 were categorized as low-risk, while 154 were classified as high-risk. No statistically significant differences were observed between MetS-Sz groups in terms of sex, smoking status, physical activity, or marital status. However, the prevalence of chronic diseases was significantly higher in the group with a high MetS-Sz score (*P* < .05; Table 2).

**Table 2:** Comparison of categorical data according to MetS groups.

		MetS-Sz groups n (%)		*P*
		Low	High	
Gender	Female	24 (92.3)	143 (92.9)	.92
	Male	2 (7.7)	11 (7.1)	
Smoking	Yes	2 (7.7)	16 (10.4)	.67
	No	24 (92.3)	138 (89.6	
Chronic disease	Yes	13 (50)	110 (71.4)	.03*
	No	13 (50)	44 (28.6)	
Physical activity	Use	5 (19.2)	32 (20.8)	.86
	Not use	21 (80.8)	122 (79.2)	
Marital status	Marriage	21 (80.8)	135 (87.7)	.34
	Single	5 (19.2)	19 (12.3)	

SD = standard deviation.

* The statistically significant difference was accepted as *P* < .05.

Significant differences were observed between the MetS-Sz groups in terms of body mass index (BMI), body fat mass, body muscle mass, body fat ratio, and body muscle ratio. Age, BMI, body fat mass, body muscle mass, body fat ratio, and body muscle ratio were found to be statistically significantly higher in the group with higher MetS-S scores (Table 3).

**Table 3:** Comparison of numerical data according to MetS groups.

	MetS-Sz groups (mean ± SD)		
	Low	High	*P*
Age	41.31 ± 11.37	46.31 ± 11.67	.044*
BMI	33.72 ± 3.76	41.34 ± 6.67	.001*
Fat mass	33.18 ± 6.06	45.20 ± 10.96	<.001*
Muscle mass	52.30 ± 8.19	58.98 ± 8.40	<.001*
Fat percentage	37.54 ± 2.56	41.74 ± 4.65	<.001*
Muscle percentage	33.19 ± 6.05	45.20 ± 10.98	<.001*

SD = standard deviation.

* The statistically significant difference was accepted as *P* < .05.

Age, BMI, and chronic disease variables that showed significant differences between the MetS-S groups were included in the regression analyses as covariance factors. Binary logistic regression was performed using parameters that differed between the MetS-Sz groups as independent variables. The effects of fat mass, muscle mass, fat percentage, muscle percentage, age, BMI, and chronic disease on MetS-Sz were analyzed.

The model was found to be significant, as indicated by Nagelkerke’s R-squared value exceeding 0.2 in the analysis where fat mass, muscle mass, fat percentage, muscle percentage, and age were included as independent variables. Additionally, the model remained significant when age, BMI, and chronic disease were included as independent variables. The model’s goodness of fit was deemed acceptable, as the *P* values from the Hosmer–Lemeshow test were >.05. Again, significant changes in the −2 Log Likelihood values between Step 1 and Step 2 (Chi-square, *P* < .05) further support the validity of the logistic regression analysis. Consequently, our model accurately predicted the outcomes with a probability of 89.4% (Table 4).

**Table 4:** Distribution of data showing the validity of the logistic regression analysis.

Method = Enter		−2 Log likelihood	Omnibus tests of model coefficients		Cox and Snell, R square	Nagelkerke, R square	Hosmer and Lemeshow test	Predicted percentage
			Chi-square	*P*				
Beginning		148.662						85.6
Step 1	Fat and muscle mass, fat and muscle percentage	110.679	37.983	<.001*	0.190	0.338	0.387	86.7
Step 2	Age, BMI, chronic disease	104.680	43.982	<.001*	0.217	0.386	<0.001	89.4

* Statistical significance was set at *P* < .05.

The odds ratio for BMI was 1.288 (1.007–1.646). This indicates that for every one-unit increase in BMI, the risk of elevated metabolic syndrome severity scores increased by 1.288 times (Table 5). In contrast, no significant associations were identified between fat mass, muscle mass, fat percentage, muscle percentage, and the severity score of metabolic syndrome.

**Table 5:** Logistic regression analysis showing the relationship between MetS-Sz groups and variables of fat mass, muscle mass, fat percentage, muscle percentage, age, BMI, and chronic disease.

MetS-Sz†		*B*	SE	Wald	*P*	Risk (Odds) coefficient (Exp B)	95% CI for (Exp. *B*)	
							Lower	Upper
Step 1	Fat mass	1.753	1.847	0.901	.343	5.770	0.155	215.357
	Muscle mass	−0.155	0.251	0.381	.537	0.856	0.523	1.402
	Fat percentage	−0.461	0.619	0.556	.456	0.631	0.188	2.120
	Muscle percentage	−1.413	1.703	0.688	.407	0.243	0.009	6.855
Step 2	Age	0.026	0.024	1.218	.270	1.027	0.980	1.076
	BMI	0.253	0.125	4.070	.044*	1.288	1.007	1.646
	Chronic disease^[1]^	−0.152	0.549	0.077	.782	0.859	0.293	2.517
	Constant	5.282	23.145	0.052	.819			

*B* = estimated coefficients, BMI = body mass index, CI = confidence interval, SE = standard error.

* Logistic regression analysis is significant at *P* < .05 level (2-tailed).

† Dependent variable: MetS-Sz.

## 4. Discussion

The findings of this study provide valuable insights into the relationship between the MetS-S and body composition, along with various demographic variables such as age, gender, and the presence of chronic diseases. However, the fact that the majority of participants were female, only 26 had low MetS-S scores, and the population primarily consisted of individuals with a BMI above 30 presents important limitations. Future research should address these limitations by incorporating a more homogeneous and larger sample size, which could yield stronger evidence to support the conclusions of this study.

In our study, individuals with elevated MetS-S scores demonstrated statistically significant increases in age, BMI, chronic disease status, body fat mass, body muscle mass, body fat ratio, and body muscle ratio. These findings are consistent with existing literature that identifies age and BMI as critical risk factors for the development of metabolic syndrome.^[9,10]^ Furthermore, epidemiological research consistently underscores the strong association between obesity and MetS.^[11]^ Additionally, the presence of chronic diseases significantly exacerbates the risk of developing metabolic syndrome.^[12]^

The regression model, which included age, BMI, and chronic disease variables that differed between MetS-S groups as independent factors, exhibited a predictability rate of 89.4%. This high rate supports the validity of our regression model. Specifically, for each one-unit increase in BMI, the risk of an elevated metabolic syndrome severity score increases by 1.288 times. Numerous studies have explored the relationship between BMI and metabolic syndrome, with findings indicating that BMI significantly increases the risk of developing metabolic syndrome.^[13]^ It have been suggested to be a dominant factor in the development of metabolic syndrome.^[14]^ In addition, BMI is a critical parameter used to calculate the metabolic syndrome severity score.^[7]^

In our analysis of the relationship between body fat mass, muscle mass, fat percentage, muscle percentage, and MetS-S scores, we did not find a statistically significant association between these variables. However, the literature presents mixed results regarding the effects of fat and muscle ratio on the risk of metabolic syndrome. Some studies have indicated a positive association between body fat mass and increased metabolic risk factors, whereas others refute this connection.^[15,16]^ Additionally, one study suggested that body muscle mass and ratio may have a protective effect on metabolic health and reduce mortality rates.^[17]^ Other studies indicate that assessing body fat may be more effective than BMI in predicting metabolic risk factors and cardiometabolic diseases.^[18]^ The metabolic syndrome severity score is a tool developed to evaluate the risk of cardiovascular disease, and investigating its relationship with body composition may yield more reliable results. From this perspective, our study uniquely contributes to the existing body of knowledge.

In conclusion, our findings indicate that no significant relationship was found between body composition parameters other than BMI and the metabolic syndrome severity score. This lack of association may be attributed to the demographic characteristics of our sample or to the sensitivity of the methods employed. We recommend that large-scale studies be conducted to further elucidate this relationship.

## Author contributions

**Conceptualization:** Yilmaz Sezgin, Serkan Çoşkun, Yunus Aslan, Seda Şimşek, Sümeyye Kaya.

**Data curation:** Yilmaz Sezgin, Serkan Çoşkun, Yunus Aslan, Seda Şimşek.

**Formal analysis:** Yilmaz Sezgin, Serkan Çoşkun, Yunus Aslan, Seda Şimşek, Sümeyye Kaya.

**Funding acquisition:** Yilmaz Sezgin, Serkan Çoşkun, Yunus Aslan, Seda Şimşek, Sümeyye Kaya.

**Investigation:** Yilmaz Sezgin, Serkan Çoşkun, Yunus Aslan, Seda Şimşek, Sümeyye Kaya.

**Methodology:** Yilmaz Sezgin, Serkan Çoşkun, Yunus Aslan, Seda Şimşek, Sümeyye Kaya.

**Project administration:** Yilmaz Sezgin, Serkan Çoşkun, Yunus Aslan, Seda Şimşek, Sümeyye Kaya.

**Resources:** Yilmaz Sezgin, Serkan Çoşkun, Yunus Aslan, Seda Şimşek, Sümeyye Kaya.

**Software:** Yilmaz Sezgin, Serkan Çoşkun, Yunus Aslan, Seda Şimşek, Sümeyye Kaya.

**Supervision:** Yilmaz Sezgin.

**Validation:** Yilmaz Sezgin, Serkan Çoşkun, Yunus Aslan, Seda Şimşek, Sümeyye Kaya.

**Visualization:** Yilmaz Sezgin, Serkan Çoşkun, Yunus Aslan, Seda Şimşek, Sümeyye Kaya.

**Writing – original draft:** Yilmaz Sezgin, Serkan Çoşkun, Yunus Aslan, Seda Şimşek, Sümeyye Kaya.

**Writing – review & editing:** Yilmaz Sezgin, Sümeyye Kaya.
